# Frequent allelic losses at 11q24.1–q25 in young women with breast cancer: association with poor survival

**DOI:** 10.1038/sj.bjc.6690430

**Published:** 1999-05

**Authors:** M Gentile, K Olsen, M Dufmats, S Wingren

**Affiliations:** Department of Biomedicine and Surgery, Division of Oncology and; Department of Health and Environment, Division of Pathology and Forensic Medicine, University Hospital, Linköping, S-581 85, Sweden

**Keywords:** early onset breast cancer, young age, poor prognosis, LOH analysis, 11q24.1–q25

## Abstract

Previous studies have demonstrated that the pathological features of breast cancer are more aggressive in younger women than in their older counterparts, and that young age may be an independent marker for adverse prognosis. These findings have raised the question whether these differences are also present at the molecular level. In order to characterize the genetic alterations associated with early-onset breast cancer, 102 cases selected for age under 37 at diagnosis were examined for loss of heterozygosity (LOH) at nine different loci on chromosomes 11, 13 and 17. Ninety cases (88%), exhibited LOH for at least one marker. The D17S855 marker, intragenic in the *BRCA1* gene, showed a high proportion of LOH (63%), whereas the intragenic marker for the *TP53* gene, HP53, exhibited LOH in 43% of the cases. On chromosome 11, frequencies of LOH peaked at the D11S969 and D11S387 markers, which expressed LOH in 53% and 48% of the informative cases, whereas D11S1818, which is proximate to the *ATM* gene, exhibited an LOH frequency of 24%. A statistically significant correlation was found between LOH at the D11S387 marker and poor survival (*P* = 0.028). No such correlation was found for the adjacent D11S969 marker, located approximately 500 kb centromeric to D11S387. We conclude that one or more as yet unidentified genes, situated in chromosome bands 11q24.1–q25, could be involved in the initiation and/or progression of breast cancer in younger women. © 1999 Cancer Research Campaign


*British Journal of Cancer* (1999) **80**(5/6), 843–849
© 1999 Cancer Research Campaign
Article no. bjoc.1998.0430

### Frequent allelic losses at 11q24.1–q25 in young women with breast cancer: association with poor survival

**M Gentile[1], K Olsen[2], M Dufmats[1] and S Wingren[1]**

[1]Department of Biomedicine and Surgery, Division of Oncology and [2]Department of Health and Environment, Division of Pathology and Forensic Medicine, Faculty of Health Sciences, University Hospital, S-581 85 Linköping, Sweden

**Summary** Previous studies have demonstrated that the pathological features of breast cancer are more aggressive in younger women than in their older counterparts, and that young age may be an independent marker for adverse prognosis. These findings have raised the question whether these differences are also present at the molecular level. In order to characterize the genetic alterations associated with early-onset breast cancer, 102 cases selected for age under 37 at diagnosis were examined for loss of heterozygosity (LOH) at nine different loci on chromosomes 11, 13 and 17. Ninety cases (88%), exhibited LOH for at least one marker. The D17S855 marker, intragenic in the *BRCA1* gene, showed a high proportion of LOH (63%), whereas the intragenic marker for the *TP53* gene, HP53, exhibited LOH in 43% of the cases. On chromosome 11, frequencies of LOH peaked at the D11S969 and D11S387 markers, which expressed LOH in 53% and 48% of the informative cases, whereas D11S1818, which is proximate to the *ATM* gene, exhibited an LOH frequency of 24%. A statistically significant correlation was found between LOH at the D11S387 marker and poor survival ($P$ = 0.028). No such correlation was found for the adjacent D11S969 marker, located approximately 500 kb centromeric to D11S387. We conclude that one or more as yet unidentified genes, situated in chromosome bands 11q24.1–q25, could be involved in the initiation and/or progression of breast cancer in younger women.

**Keywords**: early onset breast cancer; young age; poor prognosis; LOH analysis; 11q24.1–q25

Several investigators have reported that breast cancer in younger women, when compared to their older counterparts, exhibits more aggressive features including larger tumour size, presence of positive lymph nodes, absence of steroid receptors and a high S phase fraction (Wenger et al, 1993; Albain et al, 1994; Walker et al, 1996). Furthermore, young age has been shown to be an independent predictor of adverse prognosis (de la Rochefordiere et al, 1993; Albain et al, 1994; Bonnier et al, 1995), a finding that has resulted in speculation that early-onset breast carcinomas may be of a biologically different origin and therefore should be regarded as a separate disease (Adami et al, 1986; Host and Lund, 1986; de la Rochefordiere et al, 1993; Chung et al, 1996).

To date, most studies concerning genetic characterization of breast cancer have not considered the age distribution of the studied patient population, hence, knowledge about possible age-dependent differentials at the molecular level is still scarce. Accordingly, the present study was undertaken with intent to investigate and characterize the genetic alterations associated with breast cancer in younger women. For this purpose, we performed loss of heterozygosity (LOH) analysis, using nine different highly polymorphic microsatellite markers located on chromosomes 11, 13 and 17. These were selected to determine the involvement of several putative tumour suppressor loci previously shown to be implicated in breast cancer.

On chromosome 11, markers mapping closely or telomeric to the recently cloned *ATM* gene (Savitsky et al, 1995) were included. Individuals who are heterozygous for the *ATM* locus, exhibit an increased sensitivity to ionizing radiation and predisposition to breast cancer (Swift et al, 1987, 1991, 1994; Sanford et al, 1990). Easton et al (1994) estimated that 3.8% of all female breast cancer cases, and as many as 8% of early-onset cases (i.e. afflicting women under the age of 40), could be due to heterozygous mutations in the *ATM* gene. The role of *ATM* in the processes associated with cell cycle control is still unclear, although Westphal et al (1997) recently suggested that the proteins expressed by *ATM* and *TP53* might cooperate in apoptosis and suppression of tumorigenesis.

The markers selected on chromosome 13 are found in close proximity to the *BRCA2* and the *RB1* genes. The protein coded by the *BRCA2* gene, has been implied to have a protective role in cell proliferation (Vaughn et al, 1996), and mutations in the gene sequence have been reported to be responsible for a large portion of hereditary breast cancer cases (Wooster et al, 1995; Phelan et al, 1996; Tavtigian et al, 1996). Numerous investigators have shown that the *RB1* gene is frequently heterozygously lost in breast cancer (Devilee et al, 1991; Andersen et al, 1992; Borg et al, 1992). The RB protein has a significant role in cell proliferation and is known to be involved in restriction-point control and $G_1$/S phase transition during the cell cycle (Sherr, 1996).

Various tumour suppressor genes located on chromosome 17 are involved in tumour development and/or progression of breast cancer. In the present study, LOH was assessed for a number of these genes, including *TP53*, *BRCA1* and *NME1*. The protein product of *TP53* plays a central role in cell proliferation, arresting the cell cycle in the $G_1$ phase to allow repair of the DNA in response to DNA damage. The *TP53* gene has been shown to be implicated in the majority of cancer forms (Nigro et al, 1989;

*Received 31 March 1998*
*Revised 4 December 1998*
*Accepted 4 December 1998*

*Correspondence to*: M Gentile

Hollstein et al, 1991; Greenblatt et al, 1994), and the incidence of *TP53* mutations in breast cancer has been found to be higher in young patients (Caleffi et al, 1994). The role of *BRCA1* in the cell cycle has not yet been elucidated, although it has been proposed that the protein of this gene may be significant for the maintenance of the integrity of the genome and that it may interact with Rad51 (Scully et al., 1997), which in turn is known to interact with p53 (Stürzbecher et al, 1996). Recently, Jensen et al (1998) demonstrated that physical interaction between BRCA1 and a novel ubiquitin hydrolase named BAP1, enhanced BRCA1-mediated cell growth suppression. Mutations in *BRCA1* and *BRCA2* are presumed to underlie the majority of inherited breast cancer cases (Miki et al, 1994; Szabo et al, 1995). The protein coded by the *NME1* gene has been reported to exhibit metastatic suppression capabilities (Leone et al, 1991, 1993), and reduced NME1 expression has been demonstrated to be significantly associated with aggressive tumour behaviour (Bevilacqua et al, 1989; Hennessy et al, 1991).

### MATERIALS AND METHODS

### Patients

The study included 102 young female breast cancer patients diagnosed in the South-East Sweden Health Care Region, between 1980 and 1993. The patients were between 24 and 36 years of age at the time of diagnosis, with a median age of 34. Survival data were available from the Cause of Death Register provided by the National Board of Health and Welfare. At the final follow-up, 38 patients were reported to be deceased due to breast cancer; the median follow-up time was 67 months. Tissue samples from archival material were obtained from the pathology departments of hospitals in Linköping, Norrköping, Jönköping and Kalmar.

### DNA isolation

Tumour sections were selected from routinely stained formalin-fixed and paraffin-embedded material. In a minority of cases the tumour sections also contained parts of normal breast parenchyme which was removed before DNA extraction. Each case was matched with normal cells from a lymph node that was free of metastasis. A slightly modified standard procedure (Shibata et al, 1989) was used for extracting the DNA. Paraffin was removed by repeated extractions with xylene, followed by washing with decreasing concentrations of ethanol. The tissue was digested with 10 mg ml$^{-1}$ proteinase K (Boehringer-Mannheim) in a digestion buffer containing 8 mM Tris–HCl (pH 8.0), 0.8 mM EDTA, 80 mM sodium chloride and 2% sodium dodecyl sulphate (SDS). Digestion was carried out at 55°C for 36 h. Remaining enzymes were inactivated by heating the samples at 95°C for 10 min. By-products of the enzymatic digestion were removed from the nucleic acids by extraction with phenol, phenol–chloroform (1:1) and chloroform. DNA was precipitated by adding 95% (v/v) ethanol to a final concentration of 65% (v/v) and sodium acetate to a final concentration of 0.1 M, and then incubating at –70°C for 1 h. The DNA was pelleted by centrifugation at 12 000 ***g*** at 0°C for 1 h and salt residues were subsequently removed by washing with 70% ethanol. Nucleic acids were repelleted by centrifuging as above for 5 min and then vacuum-dried and resuspended in sterile double-distilled water.

### LOH analysis

Nine highly polymorphic microsatellite markers were used, mapping on chromosome arms 11q, 13q, 17p and 17q (Table 1). The estimated cytogenetic order of these markers was as follows: 11-cen-D11S1818-D115969-qtr, 13-cen-D13S260-D13S267-D13S263-qtr and 17ptr-HP53-cen-NM23-H1-D17S855-qtr. Complete sequence and chromosomal localization for the markers were obtained from the GDB™ Human Genome Database (online), Johns Hopkins University, Baltimore, MD, USA, URL: *http://gdbwww.gdb.orgl*.

Polymerase chain reaction (PCR) was performed in a total reaction volume of 22 µl, containing 25–50 ng of genomic DNA, 2 mM magnesium chloride, 1 × *Taq* Polymerase buffer solution (20 mM $(NH_4)_2SO_4$, 75 mM Tris–HCl (pH 8.5), 0.1% Tween 20), 1 µM of each primer, 0.2 mM of each dNTP and 0.5 u *Taq* Polymerase (SDS/Promega). Annealing conditions were optimized specifically for each pair of primers (Table 1), but the denaturation and extension steps were the same for all markers and were performed at 94°C for 30 s and 72°C for 45 s respectively. PCR products were confirmed by agarose (2%) gel separation and ethidium bromide staining and subsequently subjected to radioactive labelling with PCR by incorporation of α-dATP$^{32}$. Labelling conditions were identical to those used for the primary PCR,

**Table 1** Chromosomal localization, average size and frequency of heterozygosity of the nine different markers. Annealing temperatures and corresponding number of cycles used for PCR amplification are also included

| Locus symbol | Chromosomal localization[a] | Size (bp)[a] | Heterozygosity (%)[a] | Annealing temperature (°C) | Number of cycles |
|---|---|---|---|---|---|
| D11S1818 | 11q22–23 | 140–170 | 70 | 55 | 35 |
| D11S969 | 11q24.1–25 | 141–149 | 76 | 55 | 40 |
| D11S387 | 11q25 | 168–196 | 85 | 53 | 35 |
| D13S260 | 13q12.3 | 158–173 | 78 | 55 | 35 |
| D13S267 | 13q12.3 | 148–162 | 69 | 62/58[b] | 10/30[b] |
| D13S263 | 13q14.1–14.2 | 145–165 | 84 | 55 | 35 |
| HP53 | 17p13.1 | 103–135 | 90 | 68[c] | 35[c] |
| NM23-H1 | 17q21.3 | ~ 106 | NA | 55 | 35 |
| D17S855 | 17q21 | 143–155 | 82 | 58/54[b] | 20/20[b] |

[a] Data was extracted from the GDB™ Human Genome Database. [b] For the D13S267 and D17S855 markers, PCR was performed at two different annealing temperatures, i.e. 10 cycles at 62°C followed by 30 cycles at 58°C and 20 cycles at 58°C followed by 20 cycles at 54°C respectively. [c] PCR was performed using two-step cycles, i.e. the annealing and extension steps were combined to a single elongated step performed at 68°C. NA, not available.

 © Cancer Research Campaign 1999

**Table 2** Frequency of LOH and frequency of death in cases with LOH and ROH observed for the different markers. The association between LOH and poor survival was evaluated using the Log-Rank Test

| Locus symbol | No. of cases with LOH/ no. of informative cases (%) | No. of deaths with LOH/ no. of cases with LOH (%) | No. of deaths with ROH/ no. of cases with ROH (%) | Association between LOH and poor survival (*P*-value) |
|---|---|---|---|---|
| D11S1818 | 13/55 (24) | 3/13 (23) | 22/42 (52) | NS[a] |
| D11S969 | 29/55 (53) | 12/29 (41) | 12/26 (46) | NS |
| D11S387 | 31/65 (48) | 15/31 (48) | 8/34 (24) | 0.028 |
| D13S260 | 21/48 (44) | 7/21 (33) | 6/27 (22) | NS |
| D13S267 | 10/42 (24) | 3/10 (30) | 8/32 (25) | NS |
| D13S263 | 27/79 (34) | 12/27 (44) | 17/52 (33) | NS |
| HP53 | 20/47 (43) | 7/20 (35) | 8/27 (30) | NS |
| NM23-H1 | 29/64 (45) | 14/29 (48) | 11/35 (31) | NS |
| D17S855 | 26/41 (63) | 10/26 (38) | 7/15 (47) | NS |

[a] NS, not statistically significant, i.e. $P > 0.05$. LOH, loss of heterozygosity; ROH, retention of heterozygosity.

except that the number of cycles was decreased to 15. The different alleles were then separated on a denaturing polyacrylamide (6%) gel containing 8 M urea, at 45 W for 2–3 h. Gels were dried and exposed on X-ray film (Cronex 4, DuPont) using intensifying screens, for 5–40 h at –70°C. The evaluation of LOH was made by visual inspection by at least two independent investigators. LOH was considered to have occurred if the signal intensity of one allele in the tumour DNA was significantly reduced, in relation to the other allele, when compared to the signal intensity observed for the alleles in the corresponding normal DNA.

### Statistical analysis

Correlation of allelic losses between pairs of markers was evaluated with the chi-square test. Survival curves were calculated according to the method of Kaplan and Meier (1958). The log-rank test was used to assess differences in patient survival between cases with loss and retention of heterozygosity at the various markers.

### RESULTS

Of the 102 cases, 90 (88%) exhibited LOH for at least one marker; 40 (39%), 48 (47%) and 58 (57%) showed LOH for markers on chromosomes 11, 13 and 17 respectively (Table 2). Autoradiographs showing LOH for the different markers are illustrated in Figure 1. The intragenic *BRCA1* marker D17S855 exhibited the highest proportion of LOH (63%) on chromosome 17, whereas a lower incidence was observed at the HP53 and NM23-H1 loci. On chromosome 13, the highest proportion of allelic losses was found at the D13S260 marker, which expressed LOH in 44% of the informative cases. A lower incidence was observed for the D13S263 and D13S267 markers. The frequency of LOH peaked at the D11S969 and D11S387 markers on chromosome 11, affecting 53% and 48% of the informative cases, respectively, while LOH at D11S1818 was only observed in 24% of the cases.

Figure 2 shows the pattern of LOH and survival among cases where data was available for all markers on each chromosome. No overlapping region of LOH was found for markers on chromosome 17, with only one case showing loss at all three markers (Figure 2A). Two patients expressed LOH at all three markers on chromosome 13, whereas the remaining cases exhibited only partial loss with no significant overlap between markers (Figure 2B). An overlapping region of LOH was found on chromosome

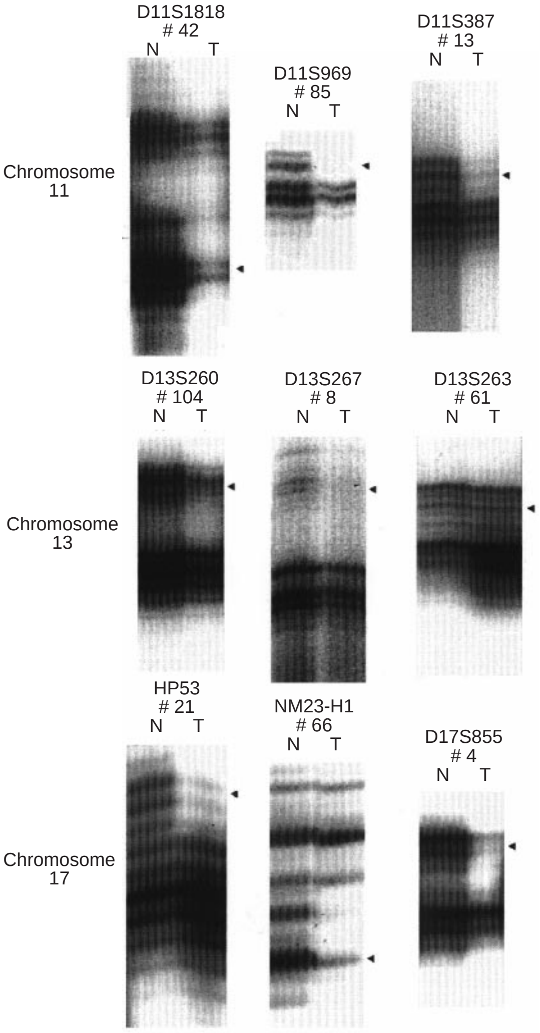

**Figure 1** Examples of autoradiographs showing allelic loss (LOH) for the nine different markers. For each case, samples containing normal DNA (N) were loaded adjacent to the matched sample with tumour DNA (T). Arrowheads indicate alleles with reduced relative intensity

© Cancer Research Campaign 1999 

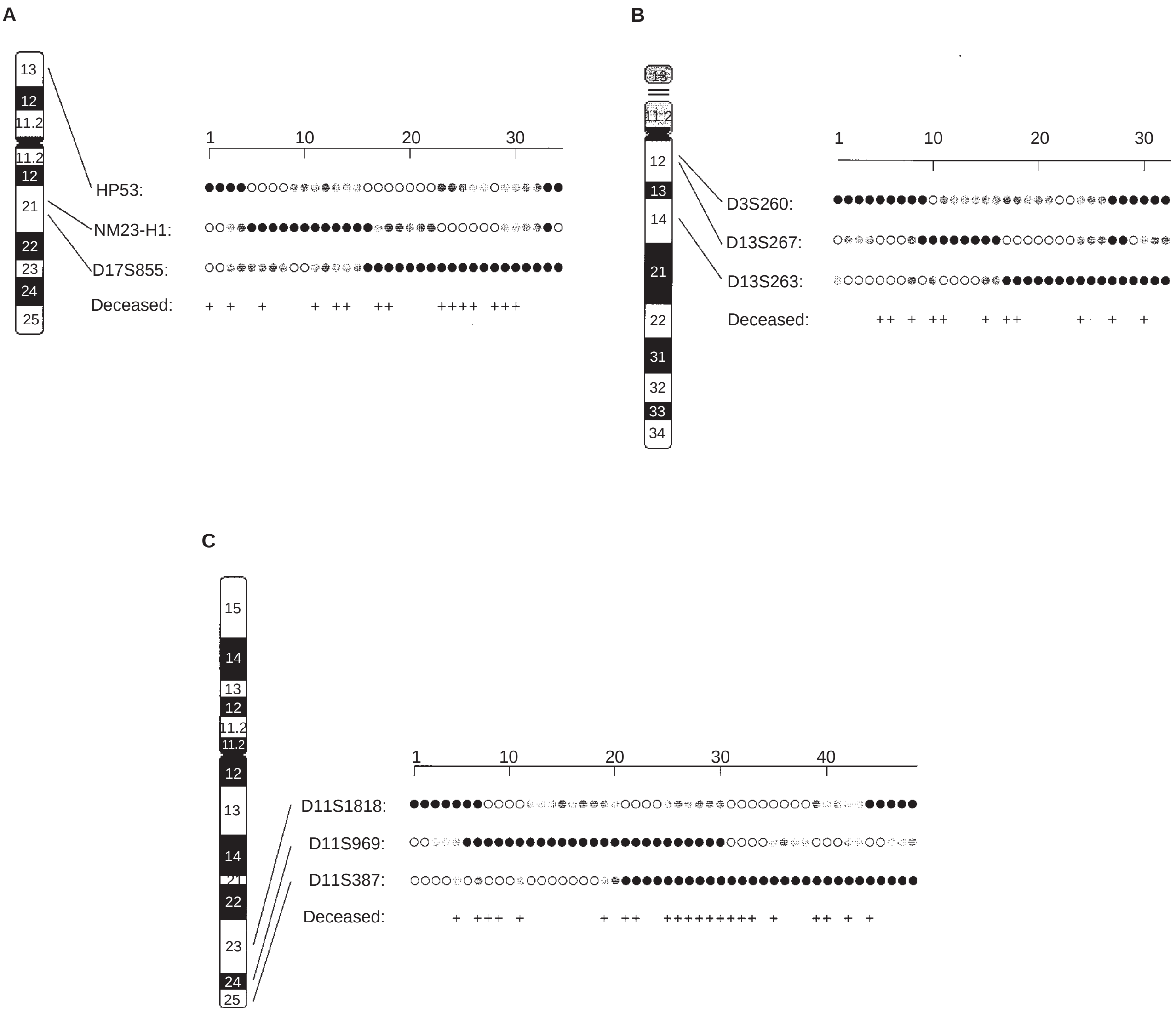

**Figure 2** Schematic representation of allelic loss in tumours where data was available for all three markers on each chromosome: **(A)** chromosome 17, **(B)** chromosome 13 and **(C)** chromosome 11. Tumours exhibiting similar pattern of LOH are grouped together. Filled black and open circles represent loss and retention of heterozygosity, respectively, whereas non-informative tumours are symbolized by filled grey circles. Cases deceased due to breast cancer are marked with a cross

11, comprising the D11S969 and D11S387 markers but excluding the D11S1818 locus (Figure 2C). Among the nine cases exhibiting LOH at the D11S387 locus but retention of heterozygosity (ROH) at the D11S969 marker, six (67%) were deceased due to breast cancer. In cases with loss of one D11S969 allele but retention of both D11S387 alleles, only two patients out of ten (20%) were found to be deceased. However, the poorest outcome, eight deaths in ten cases (80%), was observed among patients with LOH at both D11S387 and D11S969.

Log-rank test uncovered a statistically significant difference ($P = 0.028$, Table 2) in patient survival between the cases with loss and those with ROH at the D11S387 marker (Figure 3). No such correlation was found at the adjacent marker D11S969 located approximately 500 kb centromeric of D11S387, nor at markers on chromosomes 13 and 17.

Chi-square analysis performed to examine the correlation of LOH between markers at the different loci, did not unveil any statistically significant association between any combination of markers (data not shown).

### DISCUSSION

The genetic aetiology of cancer is complex and presumably proceeds through a series of alterations that affect genes at several loci on different chromosomes. In breast cancer, a number of these loci have been identified, including regions on chromosome 11, 13 and 17. The frequencies of LOH observed in the present study essentially agree with previous reports (Kerangueven et al, 1995; Nagai et al, 1995; Beckmann et al, 1996; Schmutzler et al, 1996; Kerangueven et al, 1997; Niederacher et al, 1997), and thus

 © Cancer Research Campaign 1999

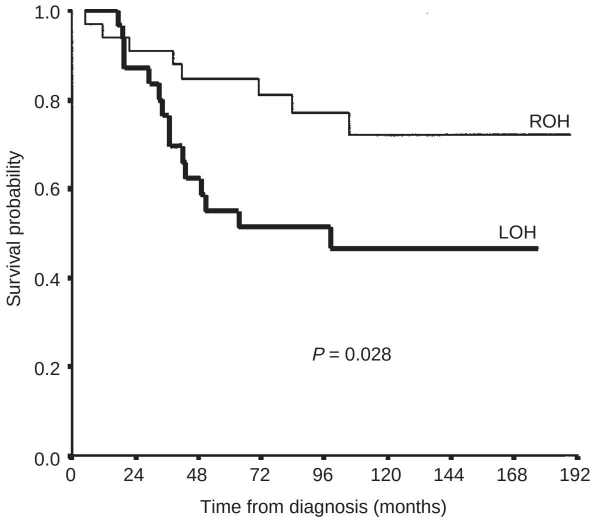

**Figure 3** Survival in relation to loss of heterozygosity (LOH) and retention of heterozygosity (ROH) at the D11S387 locus. Deaths due to other causes than breast cancer were censored. The difference in survival between the two groups was statistically significant ($P = 0.028$)

confirm that genes located in these regions may play an important role in the pathogenesis of breast cancer in young women. However, we also obtained data suggesting that a previously unidentified gene may be involved in the initiation and/or progression of early-onset breast cancer.

In keeping with several recent investigations performed on breast cancer cases not selected for young age (Gudmundsson et al, 1995; Kerangueven et al, 1997; Koreth et al, 1997), we found a high proportion of allelic losses at the telomere of chromosome 11 (Table 2). The D11S969 and D11S387 markers, located in the 11q24.1–25 region, demonstrated a significant degree of overlap with a breakpoint towards the more centromeric marker D11S1818 (Figure 2C). These findings provide support for the existence of an as yet unidentified tumour suppressor gene or genes, approximately 20 Mb telomeric to *ATM*, that may be involved in the tumorigenic process. Furthermore, log-rank analysis of our data uncovered a statistically significant correlation between LOH at the D11S387 marker and poor survival, implying that inactivation of this gene(s) may provoke more aggressive tumour behaviour. Recently, Montagna et al (1996) found evidence for the existence of a gene exhibiting sequence homology to the h-PRL-1 gene in the 11q24–q25 region. Interestingly, the h-PRL-1 gene has been suggested to play an important role in the control of basic cellular processes, such as cell growth and proliferation, making the h-PRL-1 homologue a possible candidate gene.

The proportion of LOH found at the marker for the *ATM* locus was less than half that found at the D11S969 and D11S387 markers. Moreover, as shown in Table 2, comparing the proportions of death among cases with LOH and cases with ROH between the three markers, demonstrated a two- to fourfold increase in breast cancer-specific death for the telomeric markers. These findings suggest a less important role for the *ATM* gene in early-onset breast cancer than previously postulated. In further support for our results, Fitzgerald et al (1997) recently conducted a case-control study and found that *ATM* mutations were as common in the control population as in patients with early-onset breast cancer. In addition, Vorechovský et al (1996*a*, 1996*b*) performed screening of 38 consecutive breast cancer cases, and subsequently on a larger population comprising 88 cases, for *ATM* mutations and concluded that there was no evidence for an increased number of heterozygous *ATM* carriers in the investigated population.

Compared with previous investigations of sporadic breast cancer cases not selected for age (Nagai et al, 1995; Beckmann et al, 1996; Kerangueven et al, 1997; Koreth et al, 1997), we observed a higher incidence of LOH at the D17S855 marker, which is located intragenic to *BRCA1*. This could, in part, reflect the age-dependent distribution of hereditary and non-hereditary cases, which was likely shifted towards a higher proportion of hereditary cases in the studied patient population. Marcus et al (1994) estimated that approximately half of the breast cancer cases in women under the age of 30 are of hereditary origin. It is generally recognized that *BRCA1* germ-line mutations account for almost half of the hereditary breast cancer cases (Miki et al, 1994; Easton et al, 1995), suggesting that the number of hereditary cases in the present study is probably not sufficient to satisfactorily explain the high incidence of LOH at this locus. Assuming a sequence of genetic events following Knudson's (1971) 'two-hit' hypothesis and since somatic mutations in the *BRCA1* gene appear to be an infrequent event (Futreal et al, 1994; Merajver et al, 1995; Krainer et al, 1997), a plausible interpretation of our findings could be that the surroundings of the D17S855 marker may harbour additional gene(s) that could contribute to the development and/or progression of early-onset breast cancer. It is important to note, however, that the apparent discordance observed in previous studies between *BRCA1* mutations and LOH at the corresponding locus, could imply that *BRCA1* may lose its tumour suppressor function by down-regulation caused by mechanisms other than structural mutations (Bièche et al, 1997). A recent study by Sourvinos and Spandidos (1998) confirmed this by demonstrating a two- to fivefold reduced BRCA1 expression in tumour specimen as compared to normal tissue. They proposed that the reduction in mRNA levels could be due to loss of gene copies (allelic loss), deletion of regulatory elements in the promoter region of *BRCA1* or failure in the transcriptional regulation by oestrogen receptors.

Considering the findings of several previous studies suggesting an association between *TP53* status and age (Caleffi et al, 1994; Walker et al, 1996), we anticipated the frequency of LOH at this locus to be higher than what is usually found in consecutive sporadic cases. However, the proportion of cases exhibiting LOH in the present study falls within the range of what has been previously reported for cases not selected for age (Andersen et al, 1992; Cornelis et al, 1994; Schmutzler et al, 1996; Kerangueven et al, 1997; Niederacher et al, 1997). Furthermore, we found no association between LOH and poor survival, which is somewhat surprising since Elledge and Allred (1994), in a review of the related literature, concluded that overexpression of p53 protein as well as mutations in the *TP53* gene, are independent markers for adverse prognosis in breast cancer. It is important to note though that these studies analysed *TP53* mutations or p53 protein expression, and not LOH at this locus. Although there are a few studies in which loss of *TP53* has been investigated for prognostic significance (Andersen et al, 1992; Nagai et al, 1994; Lizard-Nacol et al, 1997), the small number of cases included in these studies makes it hazardous to draw any definitive conclusions. It is thus unclear whether the lack of association between LOH at the *TP53* marker and poor survival noted in these and the present study is of any underlying biological significance, or if it merely reflects the

*© Cancer Research Campaign 1999* 

limited number of observations assessed by the statistical tests. Alternatively, the discordance between the present report and the review by Elledge and Allred could be explained by the observed LOH occurring due to alterations in gene(s) other than *TP53* residing in the 17p13.1 region.

In conclusion, it appears that one or more previously unidentified genes located in chromosomal band 11q24.1–q25 are implicated in early-onset breast cancer. Further refinement of the deleted region and eventually cloning these genes, thus enabling mutation analysis, may contribute to the understanding and elucidation of the molecular mechanisms that underlie the aetiology of breast cancer in young women.

### ACKNOWLEDGEMENTS

We would like to thank Malin Helgesson and Linda Näslund for valuable technical assistance. We also wish to acknowledge the departments of pathology at the hospitals in Linköping, Norrköping, Jönköping and Kalmar for kindly providing archival material. This work was supported by grants from the Swedish Cancer Society.

### REFERENCES

Adami HO, Malker B, Holmberg L, Persson I and Stone B (1986) The relation between survival and age at diagnosis in breast cancer. *N Engl J Med* **315**: 559–563

Albain KS, Allred DC and Clark GM (1994) Breast cancer outcome and predictors of outcome: are there age differentials? *J Natl Cancer Inst Monogr* **16**: 35–42

Andersen TI, Gaustad A, Ottestad L, Farrants GW, Nesland JM, Tveit KM and Borresen AL (1992) Genetic alterations of the tumour suppressor gene regions 3p, 11p, 13q, 17p and 17q in human breast carcinomas. *Genes Chromosomes Cancer* **4**: 113–121

Beckmann MW, Picard F, An HX, van Roeyen CR, Dominik SI, Mosny DS, Schnürch HG, Bender HG and Niederacher D (1996) Clinical impact of detection of loss of heterozygosity of BRCA1 and BRCA2 markers in sporadic breast cancer. *Br J Cancer* **73**: 1220–1226

Bevilacqua G, Sobel ME, Liotta LA and Steeg PS (1989) Association of low nm23 RNA levels in human primary infiltrating ductal breast carcinomas with lymph node involvement and other histopathological indicators of high metastatic potential. *Cancer Res* **49**: 5185–5190

Bièche I, Nogues C, Rivoilan S, Khodja A, Latil A and Lidereau R (1997) Prognostic value of loss of heterozygosity at BRCA2 in human breast carcinoma. *Br J Cancer* **76**: 1416–1418

Bonnier P, Romain S, Charpin C, Lejeune C, Tubiana N, Martin PM and Piana L (1995) Age as a prognostic factor in breast cancer: relationship to pathologic and biologic features. *Int J Cancer* **62**: 138–144

Borg A, Zhang QX, Alm P, Olsson H and Sellberg G (1992) The retinoblastoma gene in breast cancer: allele loss is not correlated with loss of gene protein expression. *Cancer Res* **52**: 2991–2994

Caleffi M, Teague MW, Jensen RA, Vnencak-Jones CL, Dupont WD and Parl FF (1994) p53 gene mutations and steroid receptor status in breast cancer. Clinicopathologic correlations and prognostic assessment. *Cancer* **73**: 2147–2156

Chung M, Chang HR, Bland KI and Wanebo HJ (1996) Younger women with breast carcinoma have a poorer prognosis than older women. *Cancer* **77**: 97–103

Cornelis RS, van Vliet M, Vos CB, Cleton Jansen AM, van de Vijver MJ, Peterse JL, Khan PM, Börresen AL, Cornelisse CJ and Devilee P (1994) Evidence for a gene on 17p13.3, distal to TP53, as a target for allele loss in breast tumors without p53 mutations. *Cancer Res* **54**: 4200–4206

de la Rochefordiere A, Asselain B, Campana F, Scholl SM, Fenton J, Vilcoq JR, Durand JC, Pouillart P, Magdelenat H and Fourquet A (1993) Age as prognostic factor in premenopausal breast carcinoma. *Lancet* **341**: 1039–1043

Devilee P, van Vliet M, Bardoel A, Kievits T, Kuipers-Dijkshoorn N, Pearson PL and Cornelisse CJ (1991) Frequent somatic imbalance of marker alleles for chromosome 1 in human primary breast carcinoma. *Cancer Res* **51**: 1020–1025

Easton DF (1994) Cancer risks in A-T heterozygotes. *Int J Radiat Biol* **66**: S177–S182

Easton DF, Ford D and Bishop DT (1995) Breast and ovarian cancer incidence in BRCA1-mutation carriers. Breast Cancer Linkage Consortium. *Am J Hum Genet* **56**: 265–271

Elledge RM and Allred DC (1994) The p53 tumor suppressor gene in breast cancer. *Breast Cancer Res Treat* **32**: 39–47

FitzGerald MG, Bean JM, Hegde SR, Unsal H, MacDonald DJ, Harkin DP, Finkelstein DM, Isselbacher KJ and Haber DA (1997) Heterozygous ATM mutations do not contribute to early onset of breast cancer [see comments]. *Nat Genet* **15**: 307–310

Futreal PA, Liu Q, Shattuck-Eidens D, Cochran C, Harshman K, Tavtigian S, Bennett LM, Haugen-Strano A, Swensen J and Miki Y (1994) BRCA1 mutations in primary breast and ovarian carcinomas. *Science* **266**: 120–122

Greenblatt MS, Bennett WP, Hollstein M and Harris CC (1994) Mutations in the p53 tumor suppressor gene: clues to cancer etiology and molecular pathogenesis. *Cancer Res* **54**: 4855–4878

Gudmundsson J, Barkardottir RB, Eiriksdottir G, Baldursson T, Arason A, Egilsson V and Ingvarsson S (1995) Loss of heterozygosity at chromosome 11 in breast cancer: association of prognostic factors with genetic alterations. *Br J Cancer* **72**: 696–701

Hennessy C, Henry JA, May FE, Westley BR, Angus B and Lennard TW (1991) Expression of the antimetastatic gene nm23 in human breast cancer: an association with good prognosis. *J Natl Cancer Inst* **83**: 281–285

Hollstein M, Sidransky D, Vogelstein B and Harris CC (1991) p53 mutations in human cancers. *Science* **253**: 49–53

Host H and Lund E (1986) Age as a prognostic factor in breast cancer [published erratum appears in Cancer 1986 Aug 15;58(4):996]. *Cancer* **57**: 2217–2221

Jensen DE, Proctor M, Marquis ST, Gardner HP, Ha SI, Chodosh LA, Ishov AM, Tommerup N, Vissing H, Sekido Y, Minna J, Borodovsky A, Schultz DC, Wilkinson KD, Maul GG, Barlev N, Berger SL, Prendergast GC and Rauscher FJ III (1998) BAP1: a novel ubiquitin hydrolase which binds to the BRCA1 RING finger and enhances BRCA1-mediated cell growth suppression. *Oncogene* **16**: 1097–1012

Kaplan E and Meier P (1958) Non-parametric estimation from incomplete observations. *J Am Stat Assoc* **53**: 457–481

Kerangueven F, Allione F, Noguchi T, Adélaïde J, Sobol H, Jacquemier J and Birnbaum D (1995) Patterns of loss of heterozygosity at loci from chromosome arm 13q suggests a possible involvement of BRCA2 in sporadic breast tumors. *Genes Chromosomes Cancer* **13**: 291–294

Kerangueven F, Eisinger F, Noguchi T, Allione F, Wargniez V, Eng C, Padberg G, Theillet C, Jacquemier J, Longy M and Sobol H (1997) Loss of heterozygosity in human breast carcinomas in the ataxia telangiectasia, Cowden disease and BRCA1 gene regions. *Oncogene* **14**: 339–347

Knudson AG Jr (1971) Mutation and cancer: statistical study of retinoblastoma. *Proc Natl Acad Sci USA* **68**: 820–823

Koreth J, Bakkenist CJ and McGee JO (1997) Allelic deletions at chromosome 11q22–q23.1 and 11q25-qterm are frequent in sporadic breast but not colorectal cancers. *Oncogene* **14**: 431–437

Krainer M, Silva Arrieta S, FitzGerald MG, Shimada A, Ishioka C, Kanamaru R, MacDonald DJ, Unsal H, Finkelstein DM, Bowcock A, Isselbacher KJ and Haber DA (1997) Differential contributions of BRCA1 and BRCA2 to early-onset breast cancer. *N Engl J Med* **336**: 1416–1421

Leone A, Flatow U, King CR, Sandeen MA, Margulies IM, Liotta LA and Steeg PS (1991) Reduced tumor incidence, metastatic potential, and cytokine responsiveness of nm23-transfected melanoma cells. *Cell* **65**: 25–35

Leone A, Flatow U, VanHoutte K and Steeg PS (1993) Transfection of human nm23-H1 into the human MDA-MB-435 breast carcinoma cell line: effects on tumor metastatic potential, colonization and enzymatic activity. *Oncogene* **8**: 2325–2333

Lizard Nacol S, Riedinger JM, Lizard G, Glasser AL, Coudray N, Chaplain G and Guerrin J (1997) Loss of heterozygosity at the TP53 gene: independent occurrence from genetic instability events in node-negative breast cancer. *Int J Cancer* **72**: 599–603

Marcus JN, Watson P, Page DL and Lynch HT (1994) Pathology and heredity of breast cancer in younger women. *J Natl Cancer Inst Monogr*: 23–34

Merajver SD, Pham TM, Caduff RF, Chen M, Poy EL, Cooney KA, Weber BL, Collins FS, Johnston C and Frank TS (1995) Somatic mutations in the BRCA1 gene in sporadic ovarian tumours. *Nat Genet* **9**: 439–443

Miki Y, Swensen J, Shattuck-Eidens D, Futreal PA, Harshman K, Tavtigian S, Liu Q, Cochran C, Bennett LM and Ding W (1994) A strong candidate for the breast and ovarian cancer susceptibility gene BRCA1. *Science* **266**: 66–71

Montagna M, Serova O, Sylla BS, Mattei MG and Lenoir GM (1996) Localization of the human phosphotyrosine phosphatase-related genes (h-PRL-1) to

© Cancer Research Campaign 1999

chromosome bands 1p35-p34, 17q12–q21, 11q24–q25 and 12q24. *Hum Genet* **98**: 738–740

Nagai MA, Pacheco MM, Brentani MM, Marques LA, Brentani RR, Ponder BA and Mulligan LM (1994) Allelic loss on distal chromosome 17p is associated with poor prognosis in a group of Brazilian breast cancer patients. *Br J Cancer* **69**: 754–758

Nagai MA, Medeiros AC, Brentani MM, Brentani RR, Marques LA, Mazoyer S and Mulligan LM (1995) Five distinct deleted regions on chromosome 17 defining different subsets of human primary breast tumors. *Oncology* **52**: 448–453

Niederacher D, Picard F, van Roeyen C, An HX, Bender HG and Beckmann MW (1997) Patterns of allelic loss on chromosome 17 in sporadic breast carcinomas detected by fluorescent-labeled microsatellite analysis. *Genes Chromosomes Cancer* **18**: 181–192

Nigro JM, Baker SJ, Preisinger AC, Jessup JM, Hostetter R, Cleary K, Bigner SH, Davidson N, Baylin S and Devilee P (1989) Mutations in the p53 gene occur in diverse human tumour types. *Nature* **342**: 705–708

Phelan CM, Lancaster JM, Tonin P, Gumbs C, Cochran C, Carter R, Ghadirian P, Perret C, Moslehi R, Dion F, Faucher MC, Dole K, Karimi S, Foulkes W, Lounis H, Warner E, Goss P, Anderson D, Larsson C, Narod SA and Futreal PA (1996) Mutation analysis of the BRCA2 gene in 49 site-specific breast cancer families. *Nat Genet* **13**: 120–122

Sanford KK, Parshad R, Price FM, Jones GM, Tarone RE, Eierman L, Hale P and Waldmann TA (1990) Enhanced chromatid damage in blood lymphocytes after G2 phase x irradiation, a marker of the ataxia-telangiectasia gene. *J Natl Cancer Inst* **82**: 1050–1054

Savitsky K, Bar-Shira A, Gilad S, Rotman G, Ziv Y, Vanagaite L, Tagle DA, Smith S, Uziel T and Sfez S (1995) A single ataxia telangiectasia gene with a product similar to PI-3 kinase. *Science* **268**: 1749–1753

Schmutzler RK, Fimmers R, Bierhoff E, Lohmar B, Homann A, Speiser P, Kubista E, Jaeger K, Krebs D, Zeillinger R, and Wiestler OD (1996) Association of allelic losses on human chromosomal arms 11Q and 16Q in sporadic breast cancer. *Int J Cancer* **69**: 307–311

Scully R, Chen J, Plug A, Xiao Y, Weaver D, Feunteun J, Ashley T and Livingston DM (1997) Association of BRCA1 with Rad51 in mitotic and meiotic cells. *Cell* **88**: 265–275

Sherr CJ (1996) Cancer cell cycles. *Science* **274**: 1672–1677

Shibata D, Brynes RK, Nathwani B, Kwok S, Sninsky J and Arnheim N (1989) Human immunodeficiency viral DNA is readily found in lymph node biopsies from seropositive individuals. Analysis of fixed tissue using the polymerase chain reaction. *Am J Pathol* **135**: 697–702

Sourvinos G and Spandidos DA (1998) Decreased BRCA1 expression levels may arrest the cell cycle through activation of p53 checkpoint in human sporadic breast tumors. *Biochem Biophys Res Commun* **245**: 75–80

Stürzbecher HW, Donzelmann B, Henning W, Knippschild U and Buchhop S (1996) p53 is linked directly to homologous recombination processes via RAD51/RecA protein interaction. *EMBO J* **15**: 1992–2002

Swift M, Reitnauer PJ, Morrell D and Chase CL (1987) Breast and other cancers in families with ataxia-telangiectasia. *N Engl J Med* **316**: 1289–1294

Swift M, Morrell D, Massey RB and Chase CL (1991) Incidence of cancer in 161 families affected by ataxia-telangiectasia. *N Engl J Med* **325**: 1831–1836

Swift M (1994) Ionizing radiation, breast cancer, and ataxia-telangiectasia [editorial; comment]. *J Natl Cancer Inst* **86**: 1571–1572

Szabo CI and King MC (1995) Inherited breast and ovarian cancer. *Hum Mol Genet* **4**: 1811–1817

Tavtigian SV, Simard J, Rommens J, Couch F, Shattuck-Eidens D, Neuhausen S, Merajver S, Thorlacius S, Offit K, Stoppa-Lyonnet D, Belanger C, Bell R, Berry S, Bogden R, Chen Q, Davis T, Dumont M, Frye C, Hattier T and Jammulapati S (1996) The complete BRCA2 gene and mutations in chromosome 13q-linked kindreds. *Nat Genet* **12**: 333–337

Vaughn JP, Cirisano FD, Huper G, Berchuck A, Futreal PA, Marks JR and Iglehart JD (1996) Cell cycle control of BRCA2. *Cancer Res* **56**: 4590–4594

Vorechovský I, Luo L, Lindblom A, Negrini M, Webster AD, Croce CM and Hammarström L (1996) ATM mutations in cancer families. *Cancer Res* **56**: 4130–4133

Vorechovský, I, Rasio D, Luo L, Monaco C, Hammarström L, Webster AD, Zaloudik J, Barbanti-Brodani G, James M and Russo G (1996) The ATM gene and susceptibility to breast cancer: analysis of 38 breast tumors reveals no evidence for mutation. *Cancer Res* **56**: 2726–2732

Walker RA, Lees E, Webb MB and Dearing SJ (1996) Breast carcinomas occurring in young women (< 35 years) are different. *Br J Cancer* **74**: 1796–1800

Wenger CR, Beardslee S, Owens MA, Pounds G, Oldaker T, Vendely P, Pandian MR, Harrington D, Clark GM and McGuire WL (1993) DNA ploidy, S-phase, and steroid receptors in more than 127,000 breast cancer patients. *Breast Cancer Res Treat* **28**: 9–20

Westphal CH, Rowan S, Schmaltz C, Elson A, Fisher DE and Leder P (1997) ATM and p53 cooperate in apoptosis and suppression of tumorigenesis, but not in resistance to acute radiation toxicity. *Nat Genet* **16**: 397–401

Wooster R, Bignell G, Lancaster J, Swift S, Seal S, Mangion J, Collins N, Gregory S, Gumbs C and Micklem G (1995) Identification of the breast cancer susceptibility gene BRCA2. *Nature* **378**: 789–792

*© Cancer Research Campaign 1999*
